# ‘LaDePa’ process for the drying and pasteurization of faecal sludge from VIP latrines using infrared radiation

**DOI:** 10.1016/j.sajce.2018.04.005

**Published:** 2018-06

**Authors:** S. Septien, A. Singh, S.W. Mirara, L. Teba, K. Velkushanova, C.A. Buckley

**Affiliations:** a Pollution Research Group, University of KwaZulu-Natal, Durban 4041, South Africa; b Chemical Engineering, University of KwaZulu-Natal, Durban 4041, South Africa

**Keywords:** Faecal sludge, Rheology, Extrusion, Drying, Infrared, Pasteurization, Reuse

## Abstract

This paper studies a faecal sludge treatment process, LaDePa (Latrine Dehydration and Pasteurization), which includes: (i) the characterization of the rheological and plastic behaviour of faecal sludge in the feeding section; (ii) the study of the drying and pasteurization performance of the process using a laboratory-scale prototype; and (iii) an evaluation of the processed faecal sludge for reuse in agriculture or as a biofuel.

Experiments conducted using a rheometer show that the faecal sludge exhibits shear thinning behaviour, i.e. viscosity decrease with shear rate increase. Plasticity tests in a cone penetrometer showed that the faecal sludge has a more liquid than plastic behaviour, which may affect extrusion quality, unless a plasticizer is added, as sawdust in this study.

The extent of drying and pasteurization of the samples was determined based on moisture content and the presence of viable Ascaris eggs respectively. As the intensity of infrared radiation was increased, drying was faster and more efficient in terms of energy consumption. However, the pellets were thermally degraded at temperatures above 200 °C. After processing in the LaDePa, Ascaris eggs were deactivated or severely damaged so that they would be not able to develop.

The last part of the study was conducted by determining the content of nutrients (C, N, P, K) and calorific value. The results showed that the processed pellets have suitable characteristics for reuse in agriculture and as a biofuel: similar nutrient content to manure and compost, and similar calorific value to wood. Drying did not affect the nutrient content and calorific value of the dry bone of faecal sludge.

## Highlights

• LaDePa is a process for the treatment of faecal sludge from pit latrine.
• Faecal sludge behaves as a shear thinning fluid and exhibits a low plasticity.
• Drying performance was higher as the infrared radiation intensity increased.
• Pasteurization of faecal sludge is achieved during the process.
• Dried sludge has suitable characteristics for reuse in agriculture or as biofuel.

## 1 Introduction

In response to the South African government commitment in 1994 to provide access to basic sanitation to citizens, the eThekwini Municipality (Durban, South Africa) has developed a plan to provide free sustainable basic sanitation (Gounden et al., 2006). After the creation of the municipality, over 30,000 ventilated improved pit (VIP) latrines were inherited from the peri-urban areas. Considered as the basic form of sanitation, VIP latrines fill up with time and are emptied in 5-years cycles at no cost to the households (Zuma et al., 2015). After pit emptying, the waste needs to be disposed in a safe and environmentally friendly way. Furthermore, the faecal sludge has the potential to be a valuable resource, provided that it is subjected to a suitable treatment (Kengne et al., 2014). For these reasons, the eThekwini Water and Sanitation in conjunction with Particle System Separation (PSS) developed the LaDePa (‘Latrine Dehydration Pasteurization’) machine for the treatment of faecal sludge from VIP latrines (Harrison and Wilson, 2012). In this process, sludge is extruded for the formation of pellets, which are then exposed to infrared radiation. The final product is dried and pasteurized pellets that are safe to handle, with minimum exposure to pathogen risk, and that are planned to be sold as an agricultural product. The use of Human excreta in agriculture can be considered as the most natural route of reuse, since it allows closing the loop of the nutrient cycle. Alternatively, the dried faecal sludge could be used as a biofuel, as proposed by Diener et al. (2014) and Muspratt et al. (2014).

Extrusion has never been studied for faecal sludge in literature. A few investigation can be found for analogue slurry materials such as manure and sewage sludge. The pelletization of manure has been conducted in order to increase its ease of handling during transportation and storage (Zafari and Kianmehr, 2012), or to mix it with lignocellulosic biomass waste prior to anaerobic digestion (Hjorth et al., 2011). The extrusion of sewage sludge mixed with clay and forest waste has been studied for brick production by Devant et al. (2011). The extrusion process requires the knowledge of the rheological, viscoelastic and plastic characteristics of the material to be extruded. In literature, only two papers from the same authors (Woolley et al., 2014a, 2014b) were found to deal with the rheology of human feacal waste. The authors showed that fresh faeces exhibit a shear thinning behaviour (i.e. decrease of viscosity with shear rate increase), and modelled it as a function of temperature and moisture content. The same shear thinning behaviour was observed for different types of manure (Hreiz et al., 2017; Liu et al., 2015; Minaei and Razavi, 2015) and sewage sludge (Cao et al., 2016; Cheng and Li, 2015; Markis et al., 2016).

Drying is an important process for the treatment and reuse of faecal and sewage sludge. The removal of moisture allows to reduce the mass and volume of the material, leading to lower costs related to the transport, handling and storage of the dried sludge. Besides, the combined effect of moisture reduction and heat in thermal drying enables the destruction of pathogen organisms. Most of pathogenic bacteria in the sludge, including Escherichia Coli, Salmonella, Shigella and Vibrio Cholerae, can be deactivated if drying achieves a moisture content with a water activity under 0.91 (Mujumdar and Devahastin, 2000). Thomas et al. (2015) found a complete destruction of Ascaris eggs at 70 °C and 6 s of residence time. Sludge drying has been studied for conventional technologies as hot air drying (Bennamoun et al., 2010; Huang et al., 2016) and contact drying (Ferrasse et al., 2002; Milhé et al., 2015), as well as emergent technologies such as superheated steam drying (Arlabosse and Blanc, 2012; Li et al., 2016), microwave drying (Bennamoun et al., 2016; Mawioo et al., 2017) and fry-drying (Ohm et al., 2017; Wu et al., 2012). In despite of the extensive applications of infrared drying, particularly in the food industry (Pawar and Pratape, 2017), no applications have been found for faecal sludge, nor sewage sludge and manure. IR radiation, defined as the electromagnetic radiation with a wavelength comprised between 0.8 and 1000 μm, has the property to transmit heat, so it can be used as a heating source during drying. Compared to conventional convective drying systems, the heat transfer during IR drying is more efficient, leading to shorter processing time, lower energy costs and more compact equipment (Nowak and Lewicki, 2004).

LaDePa process is a novel and promising technology to treat faecal sludge that has a great potential to be exported to other regions of the world, and to become a technological solution to fight against the lack of sanitation affecting 2 billion people worldwide (WHO, 2017). As new technology, there is the need to understand the process for its improvement, particularly in regards to faecal sludge extrusion and infrared drying, where literature is inexistent in the best knowledge of the authors. Likewise, the characteristics of the product have to be well known in order to be able to select an appropriate route for its reuse. Motivated on these needs, the aim of this work is to study the LaDePa process through a laboratory-scale prototype, using faecal sludge from VIP latrines collected in the eThekwini municipality. This study focuses on the fallowing targets:

• characterization of the extrusion section by the study of the faecal sludge rheological and plasticity properties;
• characterization of faecal sludge drying and pasteurization by the measurement of the moisture content, ash content and Ascaris eggs concentration at different operating conditions;
• characterization of the nutrient content (NPK) and calorific value of the processed pellets, in order to evaluate its reuse as an agricultural product or as a biofuel.

## 2 Material and methods

### 2.1 Laboratory-scale LaDePa

**Fig. 1:**
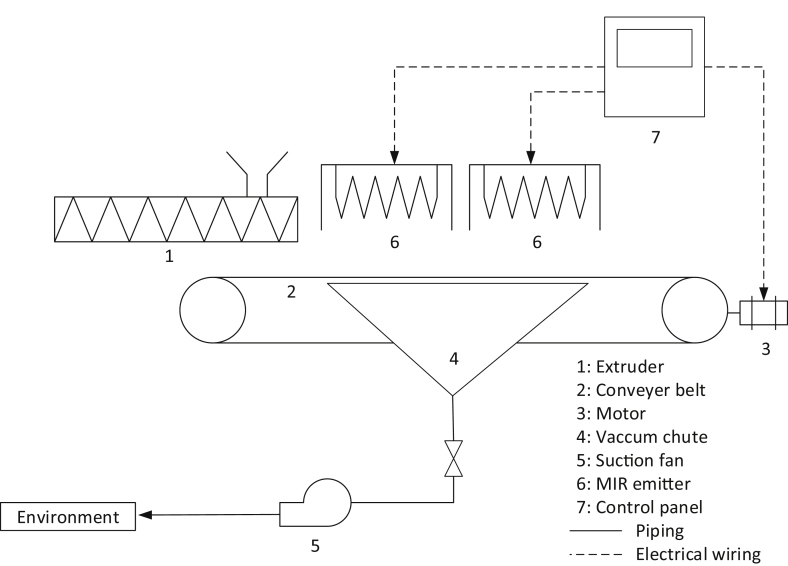
Schematic diagram of the laboratory-scale LaDePa.

A laboratory-scale LaDePa machine has been installed in the laboratory of the Pollution Research Group, at the University of KwaZulu-Natal (Durban, South Africa). This prototype is based on the full-scale LaDePa machine with a scaling factor of 1:10 and operates in a similar way. Pellets are formed by extrusion and are deposited onto the porous steel conveyer belt which transports the pellets into the heating zone. There, the pellets are exposed to heat in the form of thermal radiation from two medium wave infrared (MIR) emitters and a vacuum chute creates continuous air movement for moisture evacuation. The pellets leave the belt via a discharge chute. A schematic diagram of the laboratory-scale LaDePa is shown in Fig. 1.

### 2.2 Faecal sludge sampling and storage

Faecal sludge was sampled during pit emptying of VIPs in settlements located in the peri-urban areas of Durban, South Africa. The samples were collected from different sections of the pit, and mixed to give an average representation of the pit contents. After collection, the samples were transported to the PRG laboratory and stored in a cold room at 4 °C to preserve their properties. Before the tests, the faecal sludge was sieved through a 5.6 mm screen in order to remove the trash pieces and get a homogeneous sludge.

### 2.3 Experiments using the laboratory-scale LaDePa

The extrusion was performed with a hand held capillary extruder to produce 8 mm diameter pellets. This device consists of a tube with an air inlet connected to a pressure line on one side, and a hole on the other side. After opening the air inlet valve, the compressed air forces the faecal sludge out the hole on the opposite side. Sawdust was added to the faecal sludge before extrusion (around 4% weight), in order to produce pellets with a well-defined shape (i.e. cylinders). In the absence of sawdust, the pellets tended to flatten after extrusion. This behaviour will be discussed later in the Results and Discussion section. It was verified that the addition of sawdust does not alter the chemical and thermal properties of the feedstock.

Experiments in the laboratory-scale LaDePa was studied as a function of the MIR intensity at different residence times. This study focuses on the effect of the LaDePa process on individual pellets, so loading on the conveyer was low in order to avoid interacting phenomena. The volumetric flow rate of the suction air in the heating zone was 13 m^3^/s. A constant distance of 115 mm was maintained between the emitters and the belt. The intensities tested were 30, 50 and 80%, corresponding to a total power of approximately 3.0, 4.7 and 6.5 kW respectively. The residence time was varied from 4 to 39 min by changing the belt speed.

The experimental conditions are summarized in Table 1. The temperature in the heating zone was measured by a k-type thermocouple placed just above the belt, at the core of the heating zone. The thermocouple consists of a 1 mm wire, enveloped in thermal insulation, and a 3 mm cone at the measuring point. The irradiance was calculated from the power of the emitters and the heating area below the emitters (220 × 880 mm).

**Table 1:** Operating conditions during the experiments in the laboratory-scale LaDePa.

Run	MIR intensity [%]	Emitters power [kW]	Irradiance [kW/m^2^]	Temperature [°C]	Residence time [min]
1	30	3.0	16	85 ± 5	4
2	30	3.0	16	85 ± 5	8
3	30	3.0	16	85 ± 5	12
4	30	3.0	16	85 ± 5	17
5	30	3.0	16	85 ± 5	26
6	30	3.0	16	85 ± 5	39
7	50	4.7	24	135 ± 5	4
8	50	4.7	24	135 ± 5	8
9	50	4.7	24	135 ± 5	2
10	50	4.7	24	135 ± 5	17
11	50	4.7	24	135 ± 5	26
12	80	6.5	34	215 ± 5	4
13	80	6.5	34	215 ± 5	8

### 2.4 Characterization of the faecal sludge and processed pellets

The rheological and plastic properties of the raw sludge sample were determined in order to characterize the behaviour of faecal sludge in the screw conveyer. The moisture content and the viable Ascaris eggs concentration were measured in order to monitor the drying and pasteurization performance of the process, respectively. Measurements of volatile solids and ash content were also carried out in order to detect thermal degradation of the material. The nutrient content in terms of phosphorous, potassium, nitrogen and carbon, was analysed in order to evaluate the potential reuse of the processed faecal sludge as an agricultural product. The calorific value was determined for the evaluation of the potential use of the pellets as a biofuel. The methods for these analyses are based on standard operating procedure from the Pollution Research Group (PRG, 2014).

#### 2.4.1 Rheometer tests

The viscosity and viscoelasticity of the faecal sludge from VIP latrines were measured by a rheometer model *Anton Paar* *MCR 51*, which is the same method used by Wolley et al. (2014a, 2014b) for the analysis of fresh faeces. Two different types of tests were performed: static tests in order to measure the flow properties and dynamic tests in order to determine the viscoelastic properties of the sludge after applying a strain stress.

During the static tests, the faecal sludge was placed in a cup and a vane rotated in the sludge at increasing rotational shear rate. Through the mechanical constraints measured by the device, the shear stress and viscosity were obtained in the shear stress range of 0.001–1000 min^−1^. The total time of analysis varied between 20 and 30 min.

During the dynamic tests, the faecal sludge was again placed in a cup and the vane oscillated at different frequencies and strain stress. From the measurement of the mechanical constraints, the device can give the viscoelastic behaviour, expressed through the complex modulus with an elastic and viscous component. The viscoelastic properties were studied in the Linear Viscoelastic Region (LVR) where the complex modulus evolves linearly with the strain stress. Above this region, the sludge stops deforming and starts to flow. In this study, tests were carried out by varying the vane angular oscillatory frequency from 0.1 to 100 rad s^−1^, at a given strain stress of 1%, which was within the LVR. The total time of analysis varied between 20 and 30 min.

#### 2.4.2 Penetrometer tests

Plasticity of faecal sludge was characterized using a drop cone penetrometer with 30° apex cone. The penetrometer measures the depth of penetration of the cone from the surface of the sample, after allowing it to fall freely for 5 s. For the tests, the sample was placed in a cup of 55 mm diameter and 50 mm depth.

The Atterberg limits were determined in the penetrometer through the measurement of penetration depths, at different sample moisture contents. The moisture content leading to a penetration depth of 20 mm is called the plastic limit or liquid limit, depending on the total cone and shaft mass: 80 g for the liquid limit; 240 g for the plastic limit. In this study, the moisture content was varied by mixing the fresh sludge with dried sludge or sawdust.

#### 2.4.3 Ascaris eggs analysis

The pathogen level of the raw and dried material was measured through an Ascaris Lumbricoides analysis, performed by an external laboratory and based on the method presented by Pebsworth et al. (2012) and Naidoo (2017). In this analysis, the Ascaris eggs in the sample were classified as viable, potentially viable or dead.

Ascaris eggs were selected as indicator for this study, as they are one of the most resistant pathogens found in faecal material. If the Ascaris eggs are deactivated, it can then be assumed that the other pathogens are also deactivated.

#### 2.4.4 Moisture, volatile solids and ash content analysis

The moisture and ash content were measured for the raw faecal sludge and the pellets processed in the LaDePa apparatus at the different conditions, based on a Standard Method for the Examination of Water and Wastewater (APHA, 2012). The moisture content was determined by measuring the sample mass before and after complete drying in an oven at 105 °C for 24 h; the ash content was then determined by measuring the mass before and after calcination in a muffle furnace at 550 °C for 2 h. The volatile solids content could be then deduced by the following relationship: volatile solids content = 1 – ash content.

#### 2.4.5 Nutrient content analysis

The agricultural potential of the processed feacal sludge was evaluated through chemical analysis. The content of the nutrients, phosphorus (P) and potassium (K), were determined after digestion of the samples and analysis in a Microwave Plasma-Atomic Emission Spectrometer (MP-AES) model *Agilent 4100*. The digestion was performed by adding nitric acid to the sample and then placing the solution in a microwave digester model *Ethos 1* *–* *Milestone*, heated up to 130 °C for 1 h. The carbon (C) and nitrogen (N) contents were measured in a CN analyzer model *LECO TrueMac*.

#### 2.4.6 Calorific value measurement

In order to evaluate the potential use of LaDePa pellets as a biofuel, the calorific value was measured in an oxygen bomb calorimeter model *Parr 6200*, using the same setup and method than Zuma et al. (2015). This device is able to measure the heat of combustion of the sample after the introduction of pure oxygen.

### 2.5 Statistical analysis

Each of the drying experiments and characterization tests were performed either in duplicates or triplicates in order to verify repeatability. The uncertainty was determined from the standard deviation of the repetitions using a t-Student distribution in a 90% confidence interval.

## 3 Results and discussion

In this section, the results obtained from the experiments conducted in the LaDePa, as well as the faecal sludge and pellet characterization, are presented and discussed.

### 3.1 Rheological and plastic properties of faecal sludge

The rheological properties of faecal sludge (viscous and viscoelastic), as well as the plastic properties, were studied in order to determine the behaviour of the sludge during the mechanical transport and extrusion in the screw extruder.

#### 3.1.1 Rheological properties

**Fig. 2:**
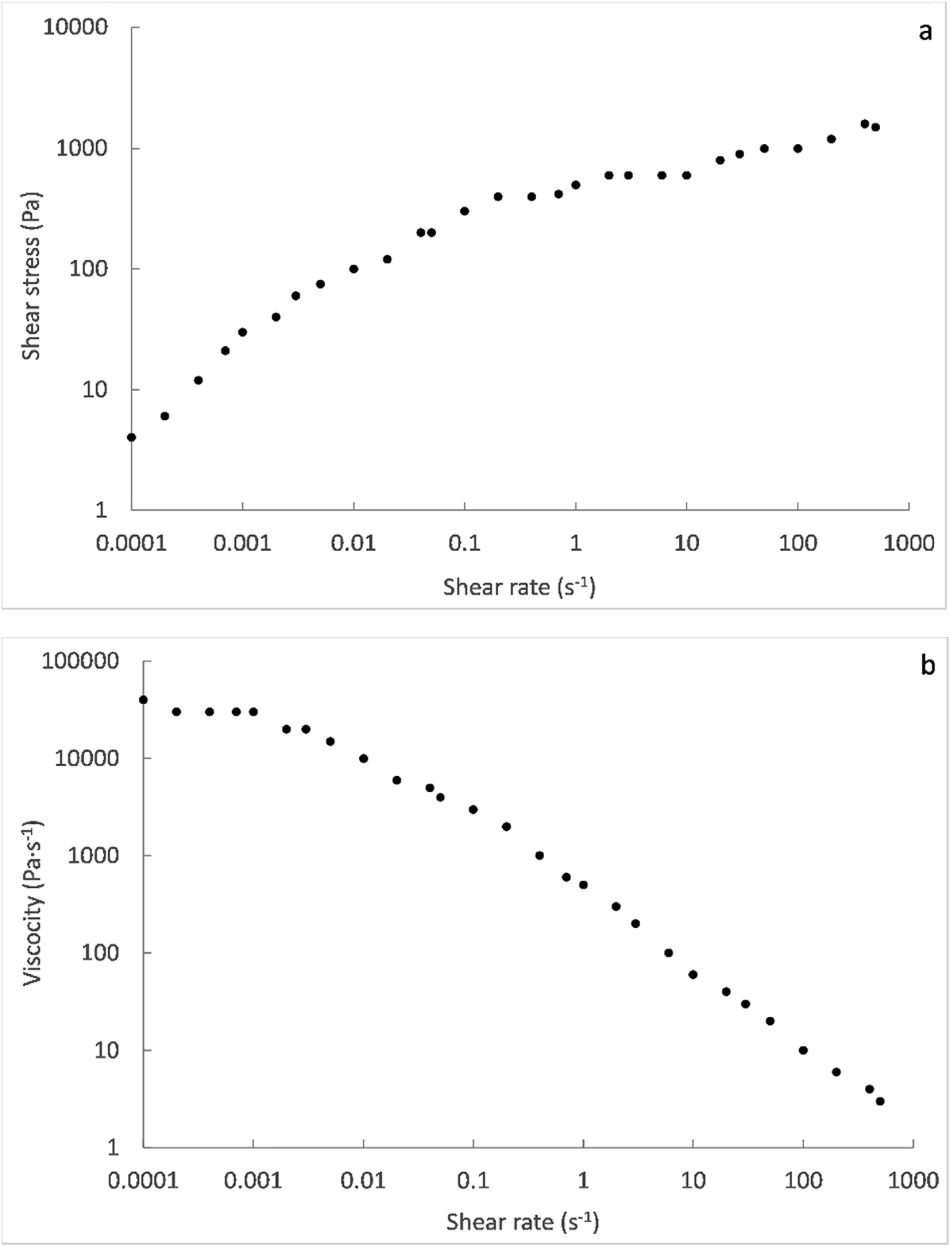
Shear stress (a) and viscosity (b) versus shear rate on logarithmic scale, for feacal sludge from VIP latrines, during static tests in the rheometer.

The flow behaviour of the faecal sludge from VIP latrines was studied through the shear stress and viscosity change as a function of shear rate. The flow curves obtained can be observed in Fig. 2.

The shear stress increased (Fig. 2a) and the viscosity decreased (Fig. 2b) by increasing the shear rate. Therefore, the sample exhibits the behaviour of a shear thinning fluid, also known as Bingham pseudo-plastic, for which the resistance to flow decreases as the applied shear stress increases. This behaviour, also observed for fresh faeces (Woolley et al., 2014a, 2014b), is typically found in slurries as manure and sewage sludge, as discussed in the introduction section.

**Fig. 3:**
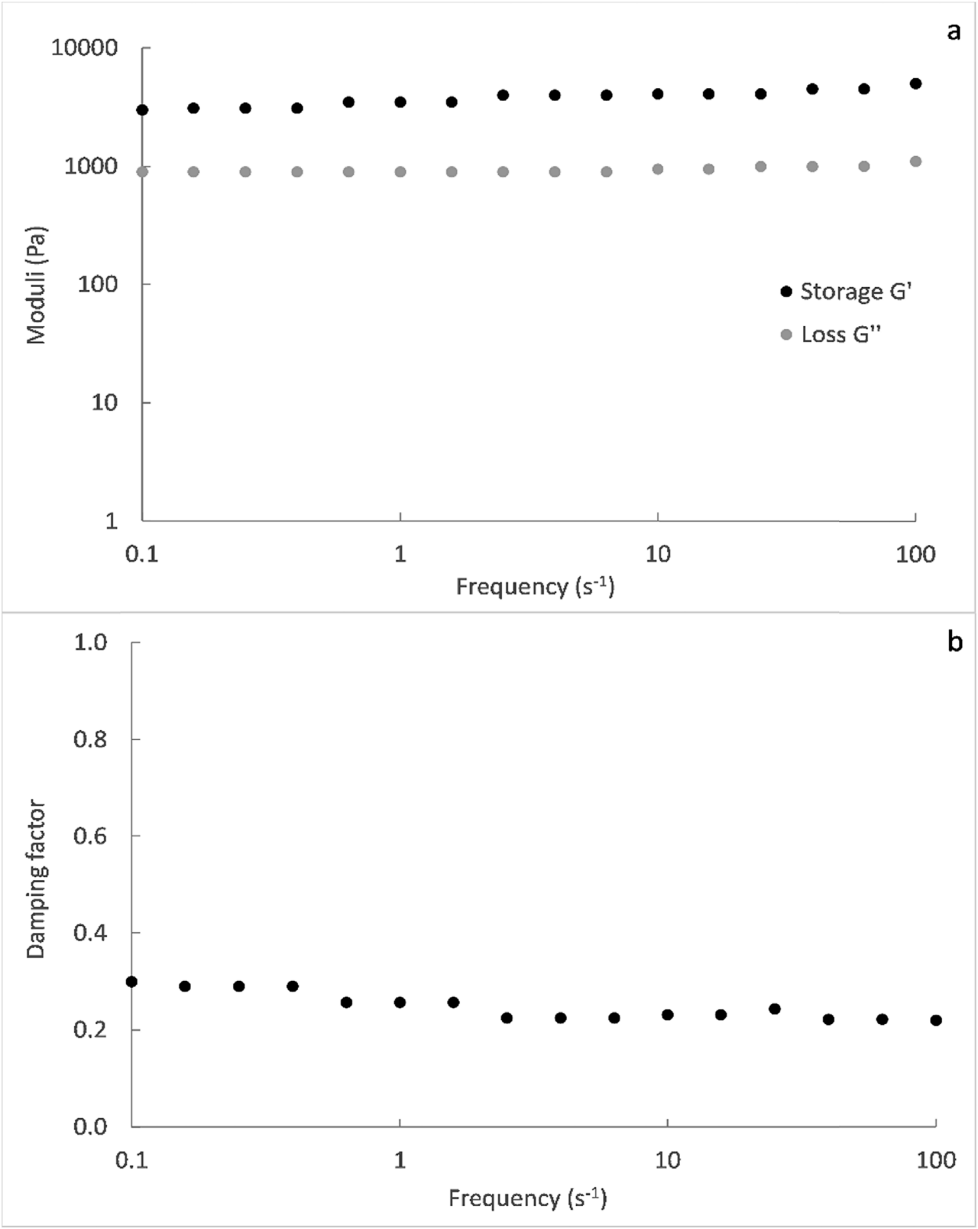
Storage and loss modulus (a) and damping factor (b) versus frequency on logarithmic scale (except for the label corresponding to the damping factor), for the feacal sludge from VIP latrines, during dynamic tests in the rheometer.

At shear rates close to zero, the shear stress presented a minimum non-zero value (Fig. 3a), which corresponds to the yield stress. The presence of a yield stress implies that a minimum shear stress, estimated at about 5 Pa, was needed for faecal sludge to flow. This behaviour could be related to the viscoelastic properties of the material. The loss and storage moduli, describing the viscous and elastic properties of the sludge respectively, remained approximately constant at 1000 and 3000 Pa respectively, in the entire frequency range tested during the dynamic tests (Fig. 3a). The damping factor, which expresses the ratio of loss to storage moduli, was considerably lower than 1 and fell in the range of 0.2–0.4 (Fig. 3b). Hence, the feacal sludge exhibits a more dominant elastic than viscous behaviour at low shear stress. For this kind of material, it is then necessary to apply a minimum shear rate in order to overcome the elastic resistance and to induce a flow. Below the yield stress, the faecal sludge deforms mainly in an elastic way, so that it reverts to its initial state after removing the strain.

#### 3.1.2 Plastic properties

Table 2 displays the liquid and plastic limits determined from two methods to lower the sample moisture content for the penetrometer tests: mixing with dried sludge and mixing with sawdust.

**Table 2:** Liquid and plastic limits obtained from penetrometer tests (moisture content in wet basis).

	Liquid limit	Plastic limit
Feacal sludge mixed with dried sludge	66.5%	61.2%
Feacal sludge mixed sawdust	73.2%	71.1%

For faecal sludge without sawdust, it can be noted that the moisture content of the VIP sludge (∼80% wet basis) was greatly above the liquid limit (∼70% wet basis). Hence, a liquid behaviour dominates in the raw sludge, which can be problematic for the formation of proper pellets as a plastic behaviour is rather suitable for this. This could then explain the problems faced during extrusion using the hand held extruder. As mentioned in section 2.2, the pellets were not able to maintain a cylindrical shape after extrusion and tended to flatten, which was probably due to the low plasiticity of the faecal sludge.

The liquid and plastic limits increased considerably for the mixtures with sawdust, leading to a sludge with more plastic behaviour. In addition, the addition of sawdust lowered the moisture content of the sample to approximately 77% wet basis, which was close to the liquid limit determined for a mixture of sludge with sawdust (∼73% wet basis). Sawdust played then the role of a plasticizer and led to the improvement of pellet formation, as described in section 2.2.

### 3.2 Drying performance

**Fig. 4:**
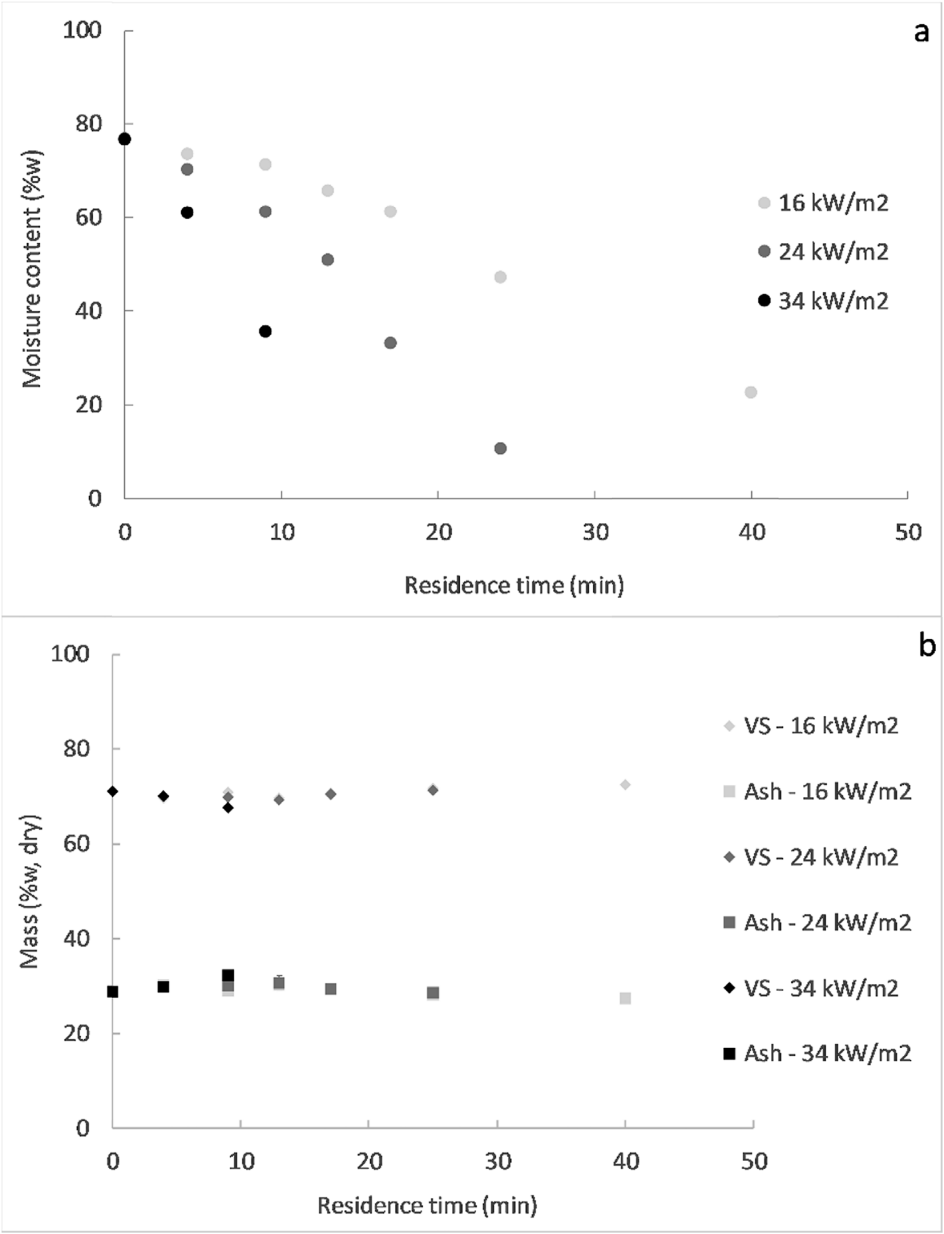
Moisture content (a), volatile solids (VS) and ash content (b), at varying residence times and MIR intensities for the 8 mm pellets.

**Fig. 5:**
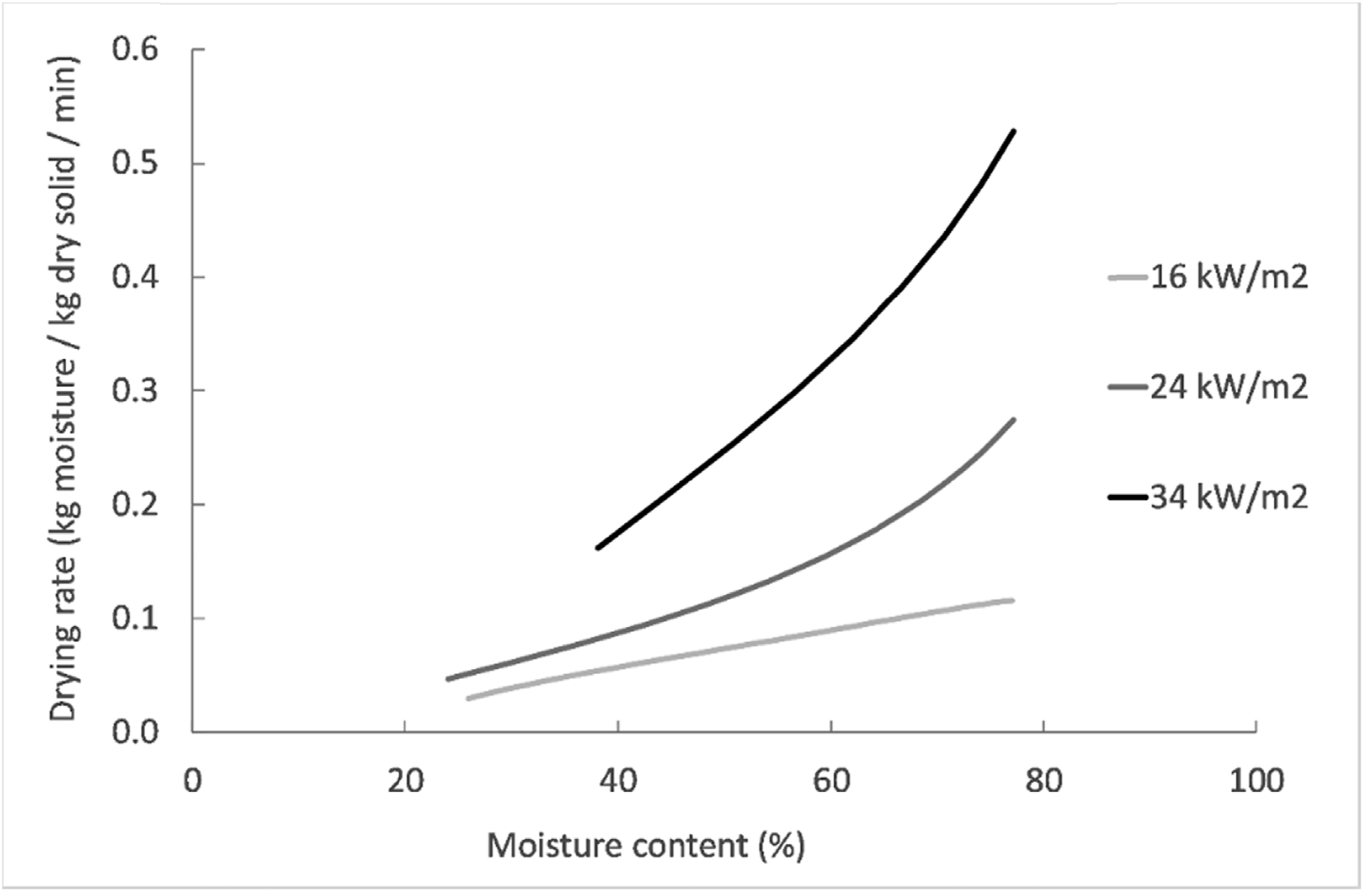
Drying rate versus moisture content at different MIR intensities.

**Fig. 6:**
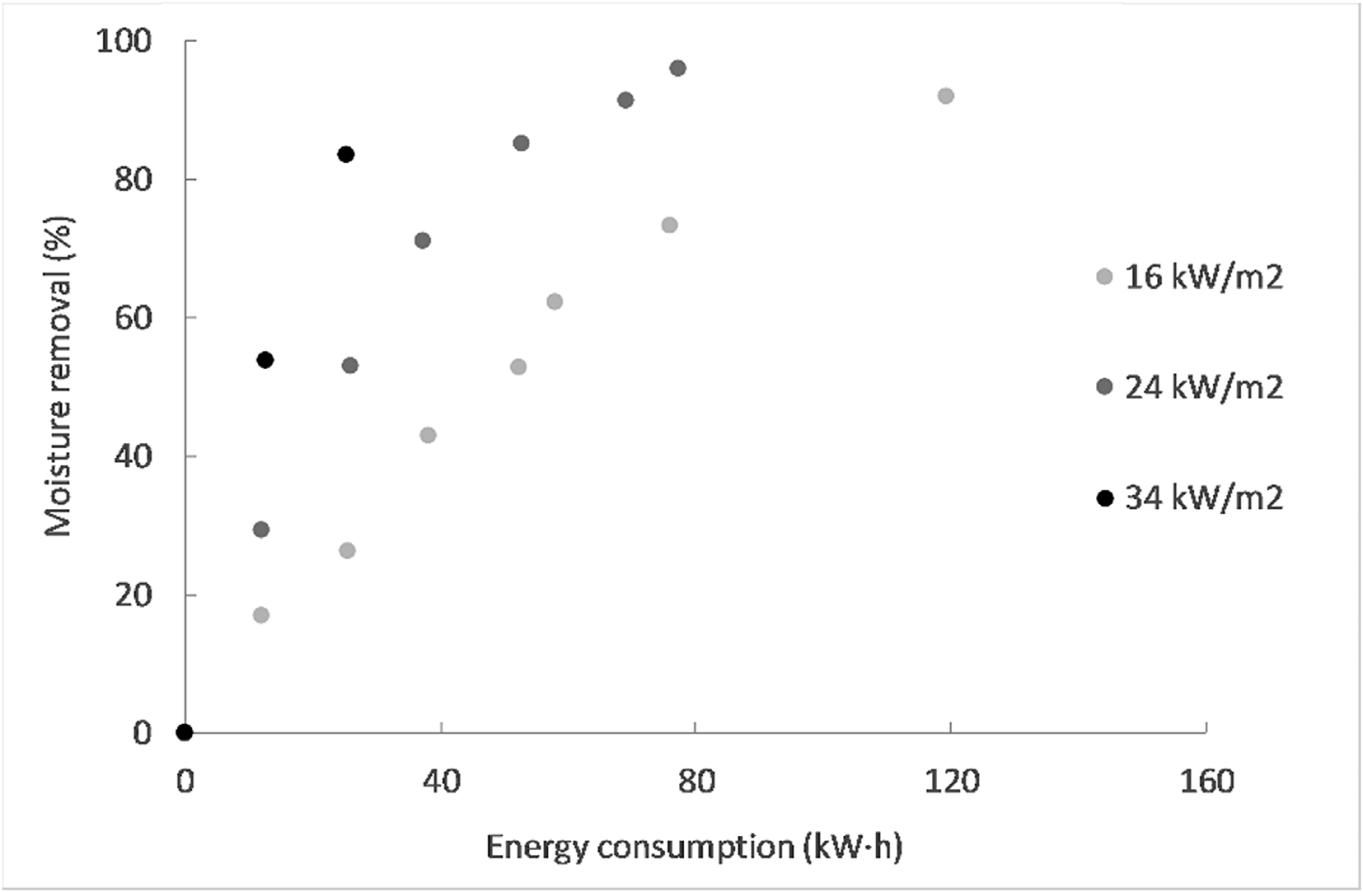
Moisture removal versus energy consumption at different MIR intensities.

Fig. 4 shows the drying curves obtained at the different MIR intensities, as well as the volatile solids and ash content evolution of the pellets during drying. Fig. 5 displays the Krischer graph, which plots the drying rates versus moisture content. The drying rates were determined after regression of the drying curve by a polynomial using an Excel function and thereafter derivation of the obtained equation with respect to time. The moisture removal versus energy consumption was plotted in Fig. 6 as a function of the MIR intensity.

As seen in Fig. 4, Fig. 5, the increase of MIR intensity led to a faster drying, as the temperature in the heating zone was higher (Table 1). At 34 kW/m^2^, the pellets were dried at the fastest rate but they were burned for residence times greater than 8 min. For that reason, no moisture content measurements could be performed after 8 min. Furthermore, the drying rate decreased with time during the process for the three MIR intensities, as it can be appreciated in Fig. 5. Drying of the pellets could then occur in the falling rate period, without a previous constant rate period. However, this assumption could be wrong since the decrease of the drying rate could be due to the reduction of the surface area of the pellets due to their shrinkage, which was observed during the experiments but not quantified. Therefore, with the available data, it is not possible to affirm or deny that drying occurred only in the falling rate period. Future investigations should be made in order to characterize the shrinkage of the sludge during experiments in the LaDePa process, which will enable to distinguish the drying rate regimes.

The volatile solids and ash content in the samples collected did not present any particular trend at varying MIR intensities (Fig. 4b). Most of the samples analysed had an ash content comprised between 0.28 and 0.32 g/g dry solid, and a volatile solid content between 0.69 and 0.72 g/g dry solid. The exception corresponded to the sample obtained at the highest heating intensity (34 kW/m^2^) after 8 min of residence time, which exhibited a slightly lower volatile solid content and slightly higher ash content. According to these results, the dry bone of the sludge seemed not to undergo considerable changes during drying, with the exception of the sample processed at the highest heating intensity after 8 min of residence time. In the latter case, the increase of the ash content and the decrease of the volatile solids content was probably caused by the beginning of the thermal degradation of the material.

The average drying rate during the experiments in the LaDePa varied between 0.07 and 1.4 g moisture/g dry solid/min. In comparison to the literature for sludges with a moisture content around 80% (similar to this work), the drying rate in this study was higher than those found from hot air convective drying between 120 and 160 °C (Bennamoun et al., 2010), contact drying at 120 °C (Ferrasse et al., 2002) and microwave drying at 3.4 kW (Mawioo et al., 2017), where the drying rates ranged between 0.01 and 0.04 g moisture/g dry solid/min. The drying rates during superheated steam and hot air drying at 160 °C (Arlabosse and Blanc, 2012) were comprised in the range from this study (∼0.1 g moisture/g dry solid/min), but they exhibited a lower value at 130 °C (0.04–0.07 g moisture/g dry solid/min). Fry-drying at temperatures between 120 and 160 °C (Wu et al., 2012) led to faster drying compared to the experiments in LaDePa, and shown drying rates comprised between 0.4 and 0.9 g moisture/g dry solid/min. These results point out that infrared drying presents high drying rates relative to other technologies.

Fig. 6 shows that the energy consumption to remove a given amount of moisture decreased by increasing the MIR intensity, thus moisture removal was more efficient at higher irradiance. In the full-scale LaDePa, the typical moisture content target to achieve is 20% wet basis. In the context of this study, the optimal drying conditions without any thermal degradation were obtained at the intermediate MIR intensity (24 kW/m^2^, ∼135 °C), where drying to 20% wet basis took less than 25 min and required around 70 kW h. At the highest MIR intensity (34 kW/m^2^, ∼215 °C), the pellets could not achieve a moisture content of 20% wet basis as they were burnt prior to this. At the lowest MIR intensity (16 kW/m^2^, ∼85 °C), the moisture content was decreased to only 20% wet basis after 40 min of residence time, and consumed approximately 120 kW h. Note that these figures are merely indicative and only the trends can be applied to the full-scale process.

### 3.3 Pasteurization performance

Ascaris analysis was performed for the 8 mm pellets processed at different residence times and MIR intensities. In Table 3, the concentrations of potentially viable Ascaris eggs are displayed. Dead Ascaris eggs concentrations are not presented as their concentration was too high to obtain any significant information about the pasteurization performance of the process.

**Table 3:** Ascaris Lumbricoides egg concentration for samples processed at different conditions.

Sample	MIR intensity (kW/m^2^)	Residence time (s)	Potentially viable Ascaris eggs/g total solid
Faecal sludge	*N.A.*	*N.A.*	135
Pellets	16	4	18^a^
Pellets	16	8	13^a^
Pellets	16	17	0
Pellets	16	25	0
Pellets	24	4	5^a^
Pellets	24	8	3^a^
Pellets	24	17	0
Pellets	24	25	0
Pellets	34	4	0
Pellets	34	8	0

a Looked intermediate between viable and dead.

The raw faecal sludge contained some viable Ascaris eggs, on the contrary of the processed pellets. No viable Ascaris eggs were detected after 17 min of residence time for MIR intensities of 16 and 24 kW/m^2^, and after 4 min of residence time at 34 kW/m^2^. A few eggs with an uncommon appearance were observed in the 8 mm pellets dried at MIR intensities of 16 and 24 kW/m^2^, after 4 and 8 min of residence time. They appeared to be intermediate between viable and dead, suggesting that they may had died just before the analysis and consequently they did not show the classical dead-look.

It can be deduced that LaDePa can lead to the deactivation of Ascaris eggs which are then unable to develop. Full deactivation was ensured after a residence time of 17 min or after 4 min when operating at a MIR intensity of 34 kW/m^2^. At residence times of 4 and 8 min at 16 and 24 kW/m^2^, a few Ascaris eggs may have not been destroyed during the process but were seriously damaged, possibly leading to their death at a later stage.

### 3.4 Nutrient content analysis

**Fig. 7:**
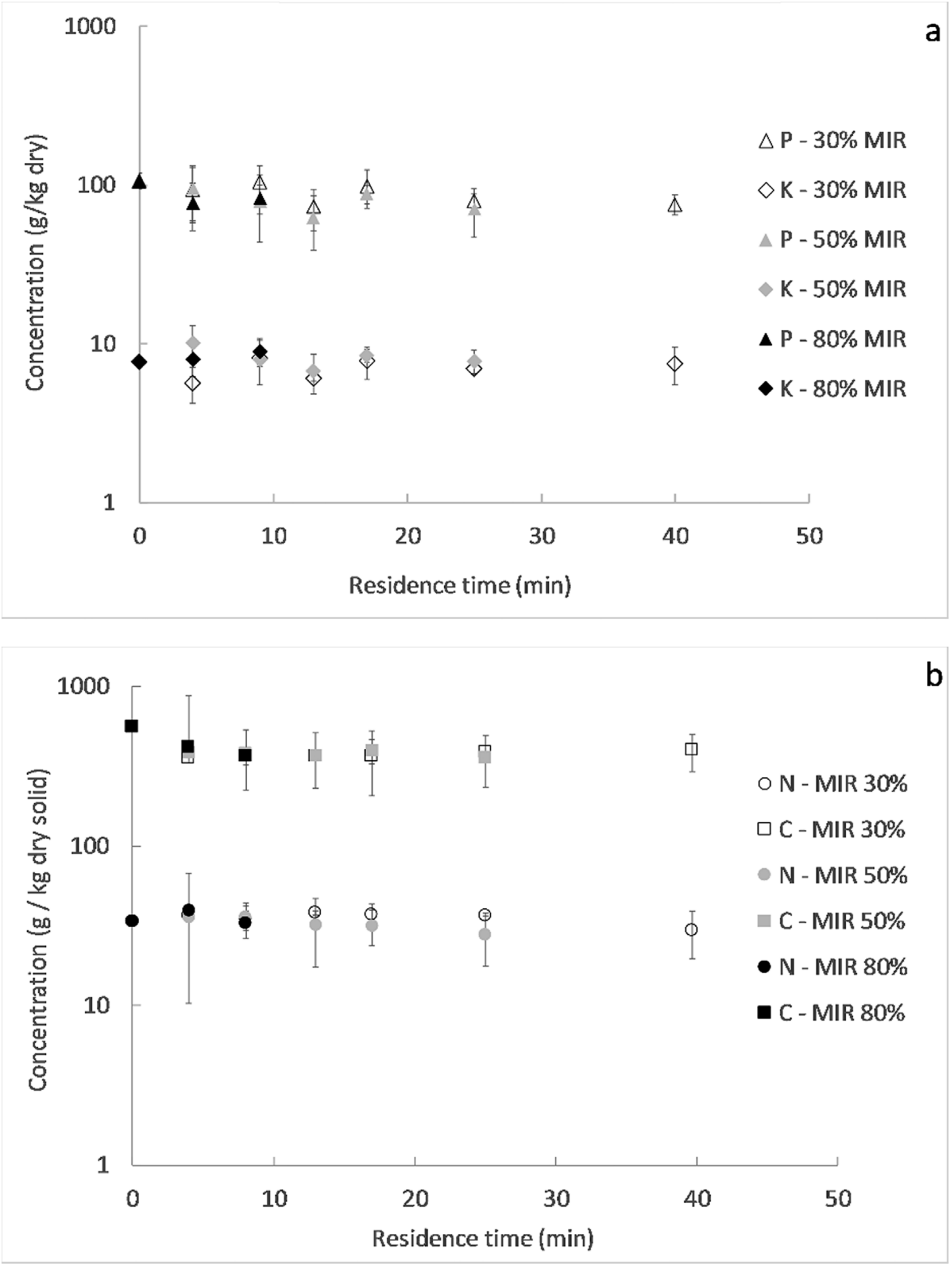
Concentration on logarithmic scale of K and P (a), and N and C (b) for the 8 mm pellets processed at different residence times and MIR intensities.

Fig. 7 shows the concentrations on dry basis of phosphorous (P), potassium (K), nitrogen (N) and carbon (C), for the pellets dried at different residence times and MIR intensities. It can be seen that the concentrations did not change significantly as a function of the operating conditions, which means that drying did not affect the P, K, Mg and Ca content in the pellets. The mean values for the P, K, N and C concentrations were approximately 85, 10, 35 and 380 g/kg dry solid respectively. As expected, C was the major constituent among the analysed elements.

The P concentration in the pellets was higher than the typical concentration from sewage sludge, manures and home compost, which varies from 0.5 to 25 g/kg dry solid (Cuevas, 1997; Ecochem, 2014; Ghaly and MacDonald, 2012; Hue and Silva, 2000; Sommers, 1977; Xu, 2014). It was in the range of some typical industrial fertilizers, e.g. ammonium phosphate sulphate (85–170 g/kg), slag basic (50–80 g/kg), superphosphate single (70–90 g/kg) and urea ammonium phosphate (55–180 g/kg). The K and N concentrations in the pellets were lower than those from usual industrial fertilizers, but were within the range of what is usually found for sewage sludge, manure and home composts: 5–25 g/kg dry solid for K; 5–50 g/kg dry solid for N (Cuevas, 1997; Ecochem, 2014; Ghaly and MacDonald, 2012; Hue and Silva, 2000; Sommers, 1977; Xu, 2014).

The N and K content of the dried pellets was similar to that of manure and compost, but with a higher P concentration. The pellets were very rich in carbon content, which represented around a third of the mass of the bone-dry solid. Based on the figures from this study, the potential annual production of nutrients in the LaDePa process would be 14.3 ton N, 35.0 ton P and 3.3 ton K, if we consider the typical production of 300 pellets per hour at a moisture content of 20% wet basis and a plant operation of 7 h during 250 working days per year. These amounts of nutrients could lead to the production of around 600 ton of maize in terms of N, 8000 ton in terms of P and 200 ton in terms of K, based on estimations of the nutrient uptake of the maize plant (Dierolf et al., 2001).

### 3.5 Calorific value

**Fig. 8:**
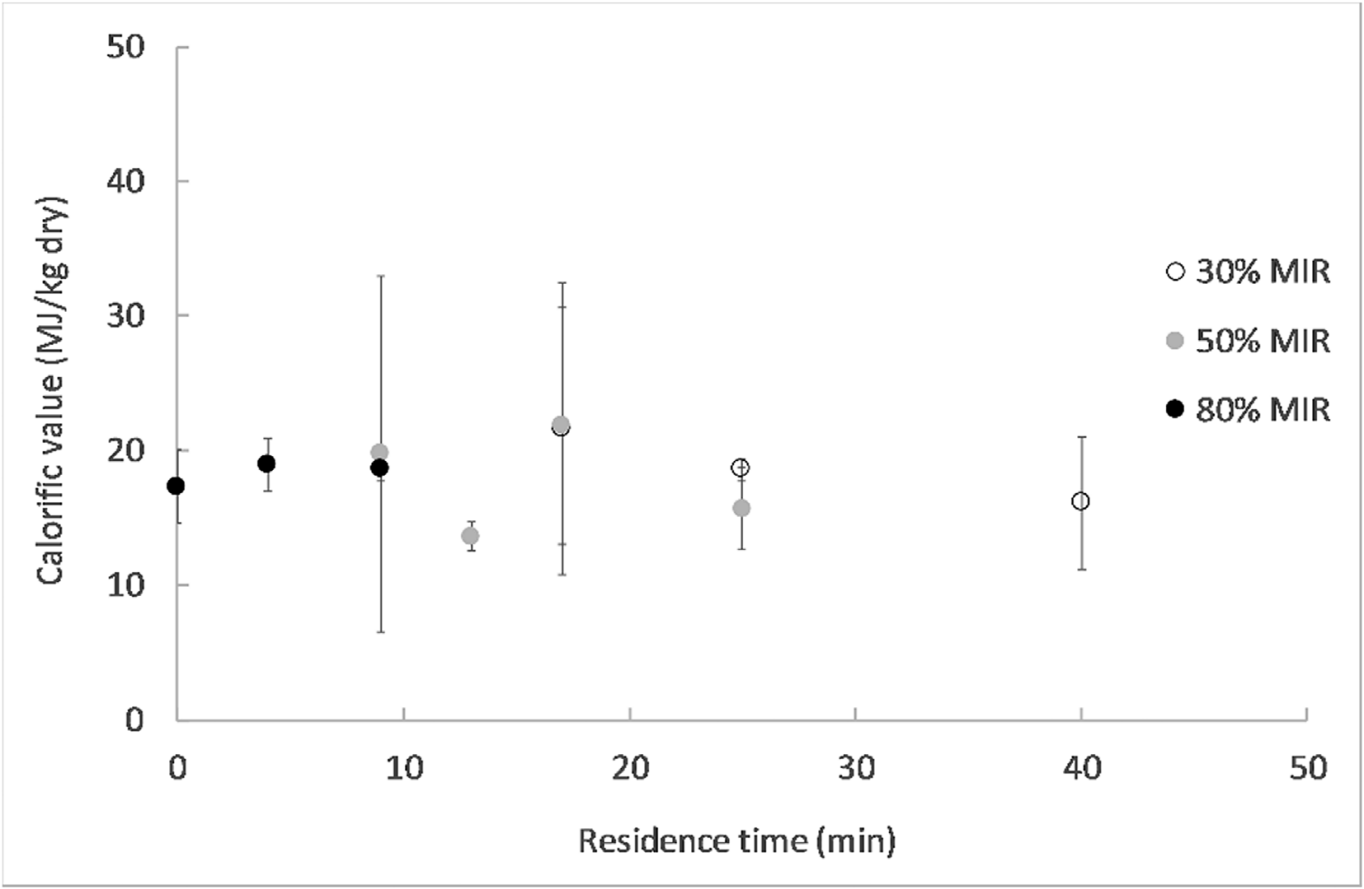
Calorific value for the 8 mm pellets at different residence times and MIR intensities.

The calorific values on a dry basis (or higher heating value) measured for the pellets dried at different MIR intensities and different residence times are shown in Fig. 8.

The calorific value of the material was not affected after drying in LaDePa. The average value, 18 MJ/kg dry solid, was similar to the calorific value of wood and some coal ranks as lignite, bituminous coal and peat (14–25 MJ/kg), as well as sewage sludge with calorific values in the range of 10–20 MJ/kg dry solid (Barz, 2009; Thipkhunthod et al., 2006). It was approximately a half to one third of the calorific value of diesel, natural gas and coal ranks as anthracite (30–45 MJ/kg). Dried faecal sludge possesses then a suitable calorific value to be used as a biofuel.

Considering a production of 300 pellets per hour at a moisture content of 20% wet basis and a plant operation of 7 h and 250 working days per year, the LaDePa process could provide biofuel with a potential energy generation of 7.7 GW h/y (power generation of 880 kW in a year average basis).

## 4 Conclusions

Faecal sludge exhibited a shear-thinning behaviour with a minimum yield stress to apply (approximately around 5 Pa), in order to overcome the elastic resistance of fluid and induce a flow. This rheological behaviour of the sludge has to be considered in the convey section from the extruder. The formation of pellets was only possible after addition of sawdust, which suggests that a plasticizer is needed for faecal sludge extrusion, due to low plastic behaviour of this material.

In the LaDePa process, the drying rate increased and the energy consumption to remove a given amount of moisture decreased by increasing the IR radiation intensity. Nevertheless, too high intensities led to undesirable thermal degradation of sludge. The optimal operation conditions must be then find at the highest MIR intensity without leading to thermal degradation. Temperatures higher than 210 °C should be avoided, as the pellets burned after 8 min of residence time under these conditions. The pasteurization of the pellets was achieved after processing in the LaDePa. Temperatures above 80 °C and residence time higher than 4 min should ensure pasteurization.

The dried pellets exhibited an interesting nutrient composition for its reuse in agriculture as organic fertilizer. In particular, the P content was relatively high: it was comparable to some industrial fertilizers and higher than typical organic fertilizers. The N and K contents were comprised in the range of sewage sludge, manure and home compost composition. The pellets exhibited a rich carbon content and thus their use as fertilizer could enrich the soil in organic matter. Moreover, the pellets demonstrated a great potential for reuse as biofuel, with a relatively high calorific value similar to wood, sewage sludge and some coal ranks. The nutrient composition and calorific value of the dry bone of the pellets were not affected by the LaDePa process.

## Appendix A Supplementary data

Data Profile Data Profile
